# The marginalization of phenomenological consciousness

**DOI:** 10.3389/fnhum.2014.00306

**Published:** 2014-05-16

**Authors:** Ethan B. Macdonald, Amir Raz

**Affiliations:** ^1^Department of Psychology, Cognitive Neuroscience Laboratory (Raz Lab), McGill UniversityMontreal, QC, Canada; ^2^Clinical Neuroscience and Applied Cognition Laboratory, Institute of Community and Family Psychiatry at the SMBD Jewish General HospitalMontreal, QC, Canada

**Keywords:** phenomenology, behaviorism, fMRI, neuroimaging, consciousness, Robert Shulman, critical neuroscience, neurophenomenology

From the height of his 90 years of experience, Robert G. Shulman is not just a veteran of World War II, but a world-class biophysicist with a distinguished research career spanning the California Institute of Technology, Bell Labs, and Yale University. A forerunner in the use of nuclear magnetic resonance, Shulman contributed to the study of biochemical processes, founded the Yale Magnetic Resonance Research Center, and shepherded functional Magnetic Resonance Imaging (fMRI) as a dominant tool of cognitive neuroscience. Together with Gregory McCarthy et al., Shulman authored several of the earliest fMRI studies, including the first to explicitly manipulate cognitive processes (McCarthy et al., 1993; Shulman et al., 1993). As such, his ideas concerning conscious research piqued our curiosity. We read *Brain Imaging: What It Can (and Cannot) Tell Us About Consciousness* with anticipation.

Shulman comes full circle with his book on brain imaging: although his career spanned meaningful collaborations with behavioral scientists and interactions with researchers from preclinical and clinical fields, he retreats to the comfort of the biophysical sciences. Two prominent, but related, hesitations give us pause regarding his retreat. First, he claims that a behaviorist approach to brain imaging is reasonable and pragmatic for the science of consciousness. Second, he rebuffs subjective and phenomenal forms of evidence. In this review we briefly address these two points of contention.

Shulman advocates for a behaviorist approach to cognitive science that espouses a stylized view of consciousness. He disregards the subjective for the sake of simplicity and, while he concedes that behavior cannot account for experience, he clearly posits subjective phenomena as a modest extension of observable behavior. For example, Shulman relies on acts of consciousness, which consist of “a person's ability to recognize and decide upon things” (p. 23) to support his ideas. Despite this description, he defines these acts of consciousness (and consciousness itself) by observable behaviors and physical brain correlates (see p. 22) rather than by the capacity to recognize or decide. This conundrum rekindles the issue of reverse inferences (Poldrack, 2006). If a computer performed acts of consciousness as part of a Turing-like test, we would hardly regard the machine as conscious because, as Searle has already argued in the Chinese room argument, if a computer or a human cannot semantically process inputs then we cannot declare it conscious (Preston and Bishop, 2002). Shulman claims that acts of consciousness do not require semantic processing. Instead, he posits that these acts demarcate consciousness and simply require “relevant background or experience” (p. 133). However, we differ from Shulman when he claims that we must attain a plane of consciousness in order to perform acts of consciousness (p. 133). We also take issue with his assertion that behaviorism constitutes a pragmatic approach to cognitive science; for example, some scholars argue that advances in artificial intelligence, rather than neuroscience, will sooner provide causal models of human behavior (Harnad and Scherzer, 2008). Taken together, we submit that cognitive science ought not conflate behavior with consciousness.

To understand consciousness, apart from behavior, scientists may require information that Shulman discards. Similar to the emphasis Shulman places on the difference between biological and physical science when he points out the tendency for biology to favor causal models over laws (p. 32), we may discern the study of consciousness from the study of biophysics. Novel neurophenomenological frameworks are rising to the challenge, helping scientists map cognition using experiential reports, psychoanalytical methods, and hypnotic suggestion (Cusumano and Raz, 2014; Lifshitz et al., 2014). These frameworks stem from an undercurrent of cognitive science that has reliably gained momentum over decades (Varela, 1999; Gallagher and Zahavi, 2012). From a philosophical standpoint, phenomenal evidence has always been compatible with the behavioral evidence that Shulman seeks (Schellenberg, 2013). Moreover, recent experimental data have confirmed the musings of philosophers. Petitmengin and Lachaux, for example, demonstrate that phenomenology and neuroimaging can work side-by-side (2013); additional research by Petitmengin and her colleagues transcends philosophy to provide clinical applications (Petitmengin et al., 2007; Petitmengin, 2010). As science presses forward into the uncharted territory of consciousness, reverting to behaviorist methods would present a curious choice. After all, we are constantly developing new tools for experiential discovery that work alongside the pre-existing neuroimaging assays Shulman helped establish.

Despite his meaningful contributions to neuroscience, Shulman conveys trepidation bridging the gap between experience and the brain. The historical failings of cognitive science, which he references extensively (p. 75–95), serve as fodder for neuroskeptics (Uttal, 2001). In the fifth chapter, he broadly covers the history of cognitive science, illustrating the flaws in research trajectories of the past and present. Unfortunately, he hardly pays as much as a passing acknowledgement to phenomenology. His solution to the problem, instead of pressing forward, centers on rekindling behaviorism. However, by removing first-person experiences from his research methodology, albeit in the name of pragmatism, Shulman ends up studying but a subset of consciousness defined by observable behavior. Although Shulman, who draws upon the work of Bennett and Hacker (2003; p. 55), seems obliquely aware of this conundrum, he nonetheless chooses to leave it unaddressed.

Regardless of how cognitive science relates to phenomenology and behaviorism, this worthwhile read will likely inspire continued discussion. Perhaps unknowingly, Shulman and his peers juxtapose phenomenology with empirical science. Whether or not readers find such concurrence palatable, Shulman presents a well-articulated account of behaviorist brain imaging. Our reservations hinge less on his advocacy for empirical and inductive science, and more on his presupposition that phenomenal and empirical evidence remain mutually exclusive.

## Conflict of interest statement

The authors declare that the research was conducted in the absence of any commercial or financial relationships that could be construed as a potential conflict of interest.

## References

[B1] Bennett M. R. Hacker P. M. S. (2003). Philosophical Foundations of Neuroscience. Malden, MA: Blackwell

[B2] Cusumano E. P. Raz A. (2014). Harnessing psychoanalytical methods for a phenomenological neuroscience. Front. Psychol. 5:334 10.3389/fpsyg.2014.00334 24808869 PMC4010798

[B3] Gallagher S. Zahavi D. (2012). The Phenomenological Mind. London: Routledge

[B4] Harnad S. Scherzer P. (2008). First, scale up to the robotic turing test, then worry about feeling. Artif. Intell. Med. 44, 83–89 10.1016/j.artmed.2008.08.008 18930641

[B5] Lifshitz M. Cusumano E. P. Raz A. (2014). Meditation and hypnosis at the intersection between phenomenology and cognitive science, in Meditation–Neuroscientific Approaches and Philosophical Implications, eds Schmidt S. Walach H. (Cham: Springer), 211–226

[B6] McCarthy G. Blamire A. M. Rothman D. L. Gruetter R. Shulman R. G. (1993). Echo-planar magnetic resonance imaging studies of frontal cortex activation during word generation in humans. Proc. Natl. Acad. Sci. U.S.A. 90, 4952–4956 10.1073/pnas.90.11.4952 8506340 PMC46631

[B7] Petitmengin C. (2010). A neurophenomenological study of epileptic seizure anticipation, in Handbook of Phenomenology and Cognitive Science, eds Schmicking D. Gallagher S. (Netherlands: Springer), 471–499

[B8] Petitmengin C. Lachaux J.-P. (2013). Microcognitive science: bridging experiential and neuronal microdynamics. Front. Hum. Neurosci. 7:617 10.3389/fnhum.2013.00617 24098279 PMC3784800

[B9] Petitmengin C. Navarro V. Le Van Quyen M. (2007). Anticipating seizure: pre-reflective experience at the center of neuro-phenomenology. Conscious. Cogn. 16, 746–764 10.1016/j.concog.2007.05.006 17590351

[B10] Poldrack R. A. (2006). Can cognitive processes be inferred from neuroimaging data? Trends Cogn. Sci. 10, 59–63 10.1016/j.tics.2005.12.004 16406760

[B11] Preston J. M. Bishop J. M. (2002). Views into the Chinese Room: New Essays on Searle and Artificial Intelligence. New York, NY: Oxford University Press Inc

[B12] Schellenberg S. (2013). Experience and evidence. Mind 122, 699–747 10.1093/mind/fzt08819332217

[B13] Shulman R. G. Blamire A. Rothman D. McCarthy G. (1993). Nuclear magnetic resonance imaging and spectroscopy of human brain function. Proc. Natl. Acad. Sci. U.S.A. 90, 3127–3133 10.1073/pnas.90.8.3127 8475050 PMC46253

[B14] Uttal W. R. (2001). The New Phrenology: The Limits of Localizing Cognitive Processes in the Brain. Cambridge, MA: The MIT Press

[B15] Varela F. (1999). Dasein's brain: phenomenology meets cognitive science, in Einstein Meets Magritte: An Interdisciplinary Reflection, eds Aerts D. Broekaert J. Mathijs E. (Brussels: Springer), 185–197

